# Expression patterns of intronic microRNAs in *Caenorhabditis elegans*

**DOI:** 10.1186/1758-907X-1-5

**Published:** 2010-02-01

**Authors:** Meltem Isik, Hendrik C Korswagen, Eugene Berezikov

**Affiliations:** 1 Hubrecht Institute, Royal Netherlands Academy of Arts and Sciences and University Medical Center Utrecht, Uppsalalaan 8, 3584CT Utrecht, The Netherlands

## Abstract

**Background:**

MicroRNAs (miRNA) are an abundant and ubiquitous class of small RNAs that play prominent roles in gene regulation. A significant fraction of miRNA genes reside in the introns of the host genes in the same orientation and are thought to be co-processed from the host gene mRNAs and thus depend on the host gene promoter for their expression. However, several lines of evidence for independent expression of intronic miRNAs exist in the literature but the extent of this independence remains unclear.

**Results:**

We performed a systematic analysis of genomic regions surrounding intronic miRNAs in the nematode *Caenorhabditis elegans *and found that, in many cases, there are extended intronic sequences immediately upstream of the miRNAs that are well-conserved between the nematodes. We have generated transcriptional green fluorescent protein reporter fusions in transgenic *C. elegans *lines and demonstrated that, in all seven investigated cases, the conserved sequences show promoter properties and produce specific expression patterns that are different from the host gene expression patterns. The observed expression patterns are corroborated by the published small RNA sequencing data.

**Conclusions:**

Our analysis reveals that the number of intronic miRNAs that do not rely on their host genes for expression is substantially higher than previously appreciated. At least one-third of the same-strand intronic miRNAs in *C. elegans *posses their own promoters and, thus, could be transcribed independently from their host genes. These findings provide a new insight into the regulation of miRNA genes and will be useful for the analysis of interactions between miRNAs and their host genes.

## Background

MicroRNAs (miRNA) are ~22 nucleotide (nt) single-stranded RNA molecules that originate from hairpin precursors and regulate gene expression at the post transcriptional level by basepairing with target messenger RNA (mRNA) and blocking its translation or inducing its degradation (reviewed in [1]). In specific cases, miRNAs can also stabilize target mRNAs [2] or even activate their translation [3]. Substantial progress has been made in recent years in the understanding of miRNA biogenesis process (reviewed in [4]). Most miRNA genes are transcribed by RNA polymerase II as long primary transcripts, or primary (pri)-miRNAs [5,6], but some miRNAs can be also transcribed by RNA polymerase III [7]. The pri-miRNA transcripts fold into stem-loop structures that are recognized and cleaved in the nucleus by RNase III-type nuclease Drosha [8,9] to release precursor miRNA hairpins (pre-miRNAs). Drosha functions together with the Pasha-DGCR8 co-factor, which recognizes the RNA substrate [10,11]; the Drosha-containing protein complex is called a Microprocessor. Recently, it has been shown that the Microprocessor is not only involved in miRNA biogenesis but can also directly regulate the stability of mRNAs by processing mRNA-embedded hairpins [12]. The pre-miRNAs hairpins produced by the Microprocessor are exported from the nucleus by exportin 5 [13-15] and further processed by another RNase III-type nuclease Dicer [16-20]. The strand with less stable basepairing at its 5' end in the resulting ~22 nt RNA duplex is loaded into Argonaute protein within RNA-induced silencing complex (RISC) and becomes mature miRNA, whereas the other strand, miRNA*, is degraded [21,22].

MiRNA genes are present in a genome as independent transcriptional units or embedded in introns of other genes (host genes) in a sense or antisense strand orientation [23]. While miRNAs residing in introns of genes in antisense orientation are, by definition, transcribed independently from the host gene, it has been assumed that sense-oriented intronic miRNAs are produced from the common transcript with their host genes - that is, they rely on the host gene promoters for their transcription [1] - and, thus, the expression of such miRNAs can be deduced from the expression patterns of the host genes. Indeed, a good correlation between the expression of miRNAs and their host genes has been observed in human microarray experiments [24]. Studies on the dynamics of pre-miRNA cropping by Drosha revealed that splicing is not required for the production of intronic miRNAs [25] and Drosha cleavage occurs co-transcriptionally without affecting the splicing of the host gene [26,27]. A separate type of intronic miRNAs, called mirtrons, bypasses the Drosha cropping altogether and, instead, relies on the splicing of the host gene to produce pre-miRNA molecules [28-30].

Although substantial experimental data exists to support the 'common transcript' model of biogenesis of intronic miRNAs, there is growing evidence that many sense-strand intronic miRNA are, in fact, transcribed independently from their host genes. Aboobaker *et al*. found that the *in situ *hybridization pattern of *mir-7 *miRNA in *Drosophila *is different from its host gene *bancal*: while *bancal *is expressed ubiquitously, *mir-7 *has a very specific spatiotemporal expression pattern, suggesting differences in the cis-regulation of this miRNA and the host gene [31]. Similarly, independent transcription of *Drosophila mir-281 *and its host gene ODA has recently been reported [32]. In humans, histone modification and RNA polymerase II occupation studies using ChiP-seq (chromatin immunoprecipitation) approaches, which can identify regions of transcription initiation or elongation, suggest that almost one-third of intronic miRNA have independent promoters [33-35]. Finally, regions directly upstream of the pre-miRNAs of two *C. elegans *intronic miRNAs were slow to drive the specific expression of GFP reporters in transgenic animals, demonstrating promoter capabilities of these intronic upstream sequences [36]. It remains unclear, however, whether independent transcriptions of intronic miRNAs and their host genes is an exception or a rule.

Here we perform a systematic study of sense-strand intronic miRNAs in *C. elegans *and show that all intronic miRNAs that have conserved upstream sequences can be transcribed from their own promoters and have specific and distinct expression patterns that differ from expression patterns of host gene promoters. Our results suggest that independent transcription of intronic miRNAs is a more frequent phenomenon than previously appreciated. The generated transgenic lines expand the set of *C. elegans *miRNA with known expression patterns and would be useful for further investigation of the biological roles of miRNAs in the worm.

## Results and discussion

### Many intronic miRNAs in *C. elegans *have conserved upstream sequences

**Figure 1:**
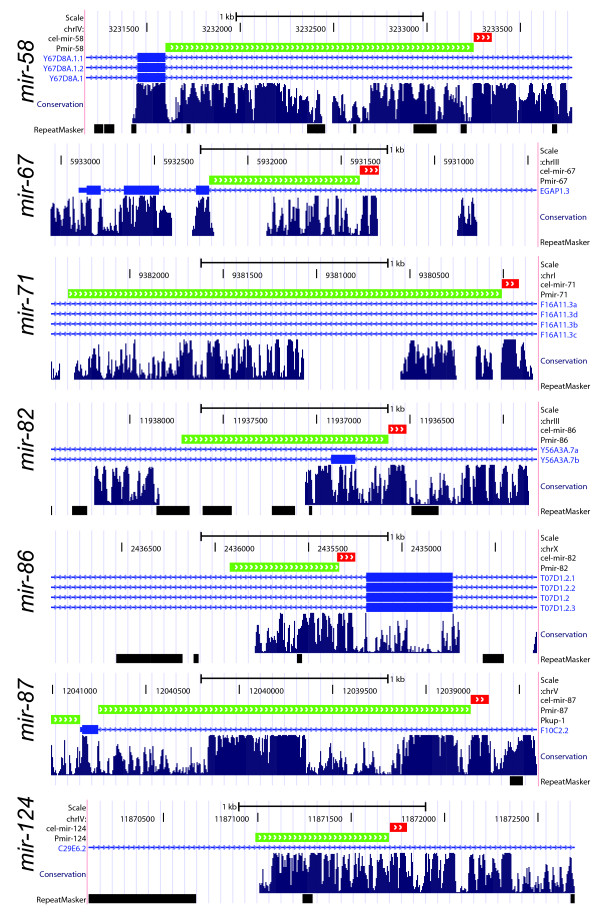
**Conservation patterns of upstream regions of intronic microRNAs (miRNA)**. The miRNA stem-loop regions are shown by red tracks, the regions selected as promoters for transcriptional *gfp *fusions are shown in green. The images are generated using UCSC genome browser [47]. Conservation tracks are based on comparison of six nematode species genomes: *Caenorhabditis elegans*, *C. briggsae*, *C. brenneri*, *C. japonica*, *C. remanei *and *Pristionchus pacificus*.

There are currently 155 annotated *C. elegans *miRNA genes (miRBase v.13), of which 103 reside in intergenic regions, 31 are embedded within an intron of a protein coding gene in a sense orientation and 21 are antisense intronic miRNAs. We first evaluated the promoter potential of upstream sequences of sense-oriented intronic miRNAs using sequence conservation between nematodes as a proxy to its functional load. From the total of 31 intronic miRNAs, 10 are located close to the exon boundary (less than 300 nt) and, thus, are less likely to posses own promoters, four miRNAs are mirtrons and five other miRNAs are probably not true miRNAs but wrongly annotated miRNA-like hairpins (Additional file 1). Of the remaining 12 miRNAs only three do not show conservation in the upstream sequences (*mir-1829b*, *mir-1829c *and *mir-1830*), while nine miRNAs have extensive conservation patterns spanning up to several hundred bases (Figure 1). The observed conservation patterns exceed an average conservation level of intronic sequences and, thus, could indicate presence of the promoter regions. Indeed, a promoter activity has been previously demonstrated for three of these regions (Table 1) - *lin-4 *[36-38], *mir-2 *and *mir-82 *[36]. The combination of observed conservation patterns and experimental evidence of promoter activity for some of the intronic miRNA upstream regions prompted us to experimentally evaluate promoter activity of the remaining intronic miRNAs with conserved upstream regions.

**Table 1:** Expression patterns of the same-strand intronic microRNAs (miRNAs) and their host genes.

miRNA	*Pmir::gfp *expression pattern	Host gene	Host gene expression pattern
*lin-4*	Expression seen from late L1 to adult stages; weak expression detected ubiquitously (except germline), stronger in pharynx, vulva, vulval muscle, body wall muscle [36-38].	F59G1.4	Expressed in head neurons: inner labial neurons, amphids and phasmids [50].
*mir-2*	Expressed from late embryos to adulthood; strong expression detected in many neurons including nerve ring, dorsal nerve cord, ventral nerve cord and also neurons in the tail [36].	*ppfr-1*	Intestine, ventral nerve cord, body wall muscle, vulval muscle, weak expression in pharynx, some neurons in head and tail [This study].
*mir-58*	Excretory canal, excretory cell soma, pharynx, intestine, hypodermis, spermatheca, absent in nervous system and in seam cells; expressed at all stages, adults have the highest expression [This study]. Note: no expression detected in [36].	Y67D8A.1	Nervous system [This study].
*mir-67*	Muscle cells (vulval, body wall and intestinal), not pharyngeal muscles; expressed at all stages, embryos and adults have the highest expression [This study].	*zmp-1*	Expressed in vulA, vulD and vulE but not vulF cells; the expression in vulD and vulE was present from late L4, whereas the expression in vulA started later in the adult [51,52].
*mir-71*	Body wall muscle, hypodermis, spermatheca, somatic gonad, excretory cell, intestine; expressed at all stages of the worm, very low expression in embryos and L2 animals, highest expression in L1s and adults [This study].	*ppfr-1*	Intestine, ventral nerve cord, body wall muscle, vulval muscle, weak expression in pharynx, some neurons in heads and tail [This study].
*mir-82*	Two amphid neurons, excretory gland cell, subset of neurons in the tail; expressed at all stages, highest expression in L4s and adults [This study]. Expression detected from L4 to adults in the pharyngeal muscle, developing spermatheca, subset of ventral nerve cord and a subset of amphid neurons [36].	T07D1.2	Early E lineage [53].
*mir-86*	Ventral nerve cord (some cells), dorsal nerve cord, subset of neurons in the tail, many neurons in the nerve ring; expressed at all stages, highest expression in L2s [This study].	Y56A3A.7	Intestine, pharynx, excretory system, somatic gonad, spermatheca, hypodermis [This study].
*mir-87*	Ubiquitous expression, strong expression in hypodermis, less expression in seam cells, anterior and posterior bulb of pharynx and nerve cord; expressed at all stages, highest expression in L4s and lowest expression in L2 [This study].	*kup-1*	Low and ubiquitous expression, also in embryos and germline [This study].
*mir-124*	Nervous system [This study].	*trpa-1*	Many tissues, including pharyngeal muscle and body wall muscle, the excretory system, the rectal gland cell, vulval epithelium, epithelial cells in the head, and the spermatheca, the majority of amphid sensory neurons and the phasmid neurons PHA and PHB, sensory neurons OLQ and IL1, other neurons in the head and ventral nerve cord [54].

### Conserved upstream sequences of intronic miRNAs have specific promoter activities

Transcriptional reporters, where promoter of interest is fused with GFP, are widely used to investigate gene expression patterns in *C. elegans *[39], and this approach was recently successfully applied to study the expression of 89 worm miRNAs. In order to investigate whether the conserved sequences upstream of intronic miRNAs can function as promoters, we similarly fused the selected regions of seven miRNAs to GFP reporters and established a number of transgenic *C. elegans *lines using a biolistic transformation [40]. In a previous miRNA promoter study Martinez *et al*. used up to 2 kb intergenic sequences upstream of pre-miRNA in order to define the promoter regions [36]. Here we restricted the promoter regions by either the upstream exon boundary of host genes or by the drop in conservation pattern, usually due to the presence of a repeat element. Regions selected for testing included five miRNAs that were not previously studied (*mir-67*, *mir-71*, *mir-86*, *mir-87 *and *mir-124*) and two miRNAs (*mir-58 *and *mir-82*) for which GFP fusions were published [36] (Figure 1 and Additional file 2).

For all of the seven investigated regions we observed distinct GFP expression patterns (Table 1) supported by at least three independent transgenic lines each. The expression pattern of *mir-82 *obtained in our study concurs with the previously published expression pattern of this miRNA [36]. In addition, we observed a very strong expression of *mir-58 *at all developmental stages in excretory cells, epidermis and intestine of *C. elegans *(Table 1), whereas Martinez *et al*. did not detect expression of the *Promoter::gfp *fusion for this miRNA [36]. The small RNA cloning data suggest that miR-58 is the most abundant miRNA expressed at all developmental stages of *C. elegans *and presumably plays a housekeeping role [41], which fits with the *Pmir-58::gfp *expression patterns observed in our transgenic lines. Interestingly, the *mir-58 *promoter region tested by Martinez *et al*. spans 2 kb upstream of pre-miRNA and includes short upstream exon and part of another intron [36], whereas the sequence used in our study is 350 bases shorter and spans the region between pre-miRNA and the upstream exon. Perhaps the presence of this exon outside of its native genomic context influenced the activity of the downstream promoter region in the previous study.

**Figure 2:**
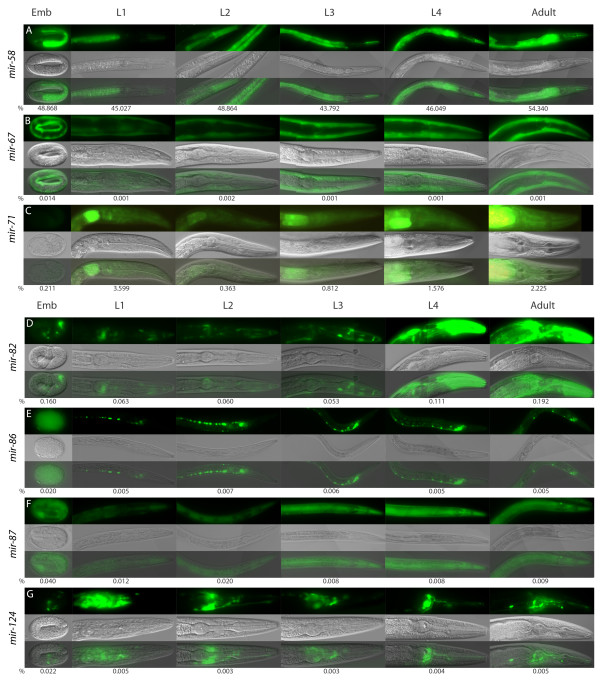
**Stage-specific changes in expression of *PmiRNA *transgenes**. The panels consist of green fluorescent protein (top), differential interference contrast (middle) and overlap (bottom) images. The representative regions are shown for different miRNAs. The numbers below the panels are drawn from small RNA cloning data by Kato *et al*. [41] and represent percentages of the miRNA reads from the total number of miRNA reads in a given developmental stage. (A) *Pmir-58*; (B) *Pmir-67*; (C) *Pmir-71*; (D) *Pmir-82*; (E) *Pmir-86*; (F) *Pmir-87*; (G) *Pmir-124*.

Promoter regions of several intronic miRNAs show tissue-specific expression: *mir-86 *and *mir-124 *are expressed only in neuronal cells and *mir-67 *is expressed only in muscle cells (Table 1). The remaining two miRNAs, *mir-71 *and *mir-87*, show broad expression patterns. Interestingly, *mir-71 *is expressed at all stages and in all cells excluding germline (Table 1), whereas *mir-2*, which resides in a different intron of the same host gene *ppfr-1*, is prominently expressed in neurons [36]. Moreover, small RNA cloning data reveals dynamic changes in expression of miR-71, with significant up-regulation at the mid-L1 stage, down-regulation at mid-L2 stage and recurrent up-regulation after mid-L4 stage [41], and our *Pmir-71::gfp *transgenic lines recapitulate this small RNA cloning pattern (Figure 2). For *mir-67*, *mir-82*, *mir-86*, *mir-87 *and *mir-124 *the relative small RNA cloning frequencies are low (less than 0.2% of total miRNA reads) but detectable at all developmental stages [41] and corroborate temporal expression patterns observed in our study (Figure 2).

### Comparison of expression patterns of host gene promoters and intronic miRNAs

**Figure 3:**
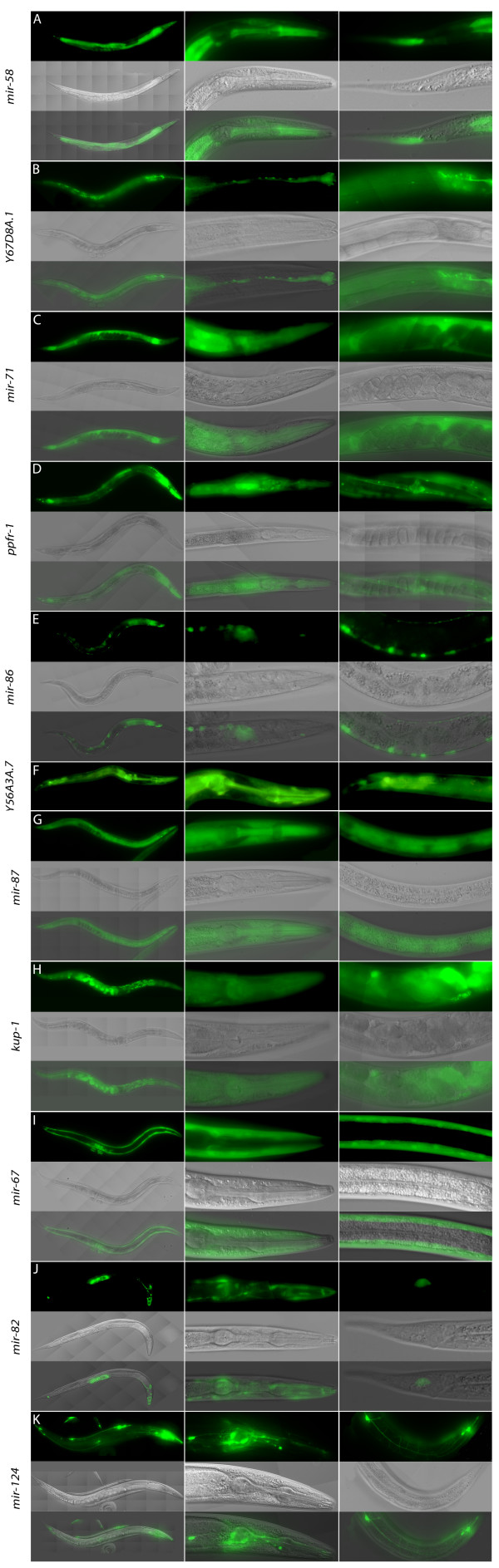
**Expression patterns of intronic microRNA promoters and their host gene promoters identified by transcriptional *gfp *fusions**. The panels consist of green fluorescent protein (top), differential interference contrast (middle) and overlap (bottom) images, and three panels in a row are shown per promoter, including whole worm and representative regions. (A) *Pmir-58*; (B) *PY67D8A.1*; (C) *Pmir-71*; (D) *Pppfr-1*; (E) *Pmir-86*; (F) PY56A3A; (G) *Pmir-87*; (H) *Pkup-1*; (I) *Pmir-67*; (J) *Pmir-82*; (K) *Pmir-124*.

We next compared the expression patterns driven by host gene promoters and predicted intronic miRNA promoters. For three of the host genes (*trpa-1*, T07D1.2 and *zmp-1*) expression patterns were already available in the literature, and for the other four genes (Y67D8A.1, Y56A3A.7, *kup-1 *and *ppfr-1*) we have generated transcriptional GFP reporter fusions (Figure 3). For some miRNA/host gene pairs we observed partially overlapping expression patterns, while for some pairs the expression patterns appeared to be completely non-overlapping (Table 1). The latter include *mir-58*, which is expressed in multiple tissues but not in the nervous system, while the host gene Y67D8A.1 is expressed only in nervous system. The *mir-86*/Y56A3A.7 pair shows an opposite pattern: *mir-86 *is expressed exclusively in the nervous system, while the host gene is expressed in the intestine, pharynx, excretory system and somatic gonad but not in neurons. The pairs with partially overlapping expression patterns include *mir-87*/*kup-1 *(ubiquitous expression), *mir-67*/*zmp-1 *(non-pharyngeal/vulval muscles) and *mir-71/ppfr-1 *(body wall muscle).

### Host gene-dependent or independent expression of intronic miRNAs?

It is recognized that the promoter fusions only approximate the expression patterns of the genes and that the actual expression can be different due to a number of factors, including incompleteness of cis-regulatory elements used in the reporters, genomic context, copy number and posttranscriptional regulation [39], in the majority of the investigated cases GFP reporters recapitulate gene expression quite faithfully [42]. In the case of miRNA genes, the expression patterns established by GFP reporters should ultimately be confirmed by *in situ *hybridization experiments. Unfortunately, miRNA *in situ *in *C. elegans *this proved to be extremely difficult and, to our knowledge, no successful procedure has so far been developed. Thus, we have used indirect evidence to investigate the expression patterns of intronic miRNA genes.

The first discovered miRNA gene, *lin-4*, was initially classified as an intergenic miRNA and shown to have a functional upstream promoter [36-38]. It was realized only recently that, in fact, this miRNA resides in a large intron of a protein-coding gene [36]. In addition, two other intronic miRNAs (*mir-2 *and *mir-82*) were recently shown to have their own promoters [36].

Here we show that many intronic miRNA genes have conserved intronic upstream sequences, which can drive specific expression of transcriptional GFP fusions in transgenic *C. elegans *animals. The observed expression patterns are only partially overlapping, or completely non-overlapping, with the expression patterns of host genes. However, the presence of functional promoters in intronic sequences does not exclude the parallel production of mature miRNAs from the host gene transcripts by the mechanisms elucidated previously (reviewed in [4]). At the same time, small RNA cloning data from various developmental stages of *C. elegans *[41] support expression patterns derived from the intronic promoters rather than from the host genes for several investigated miRNAs (*mir-71*, *mir-58*).

Many miRNA genes in *C. elegans *exist as families that share the same seed sequence (reviewed in [1]). Such miRNAs are thought to evolve by duplication of the ancestral miRNA loci followed by divergent evolution [43-46]. Interestingly, in the *mir-80 *family of miRNAs there is one intergenic (*mir-80*), one antisense intronic (*mir-81*) and one sense intronic (*mir-82*) miRNA; the sense and antisense intronic miRNAs reside in the same host gene but in the different introns. Since intergenic *mir-80 *and intronic antisense *mir-81 *should have their own promoters, and we and Martinez *et al*. [36] show that intronic sense-oriented *mir-82 *also has an intronic promoter, the most parsimonious explanation of the evolution of the *mir-80 *family is by the duplication of the locus, which included the promoter region of the ancestral miRNA. In this case, the expression patterns of *mir-80 *family members are expected to be similar and, indeed, both *mir-80 *and *mir-82 *have overlapping expression in excretory cells, head neurons and head muscles (Table 1 and [36]).

Evidence of the independent expression of intronic miRNAs and their host genes also exists in other species. In *Drosophila*, Aboobaker *et al*. demonstrated, by *in situ *hybridization experiments, different expression patterns for *mir-7 *and the host gene *bancal *[31], while Xiong *et al*. showed the independence of *mir-281 *and the host gene ODA [32]. In humans, almost one-third of intronic miRNAs are estimated to have independent promoter regions based on RNA polymerase II occupation and chromatin modification studies [33-35], although no direct promoter activity has yet been demonstrated. Thus, there is substantial combined evidence to support the independent transcription of some intronic miRNAs. At the same time, more than half of the same-strand intronic miRNAs in *C. elegans *are located in introns close to the exon boundaries (Additional file 1) and, thus, are less likely to have independent promoters but, rather, rely on host genes for their expression. We propose that such 'true' intronic miRNAs could evolve in two ways. In one scenario, an independently transcribed miRNA first becomes embedded in an intron of a host gene, in a sense or antisense orientation, and such integration in an actively transcribed genomic region could provide evolutionary advantages. In support of this 'open chromatin embedding' hypothesis, there is a comparable number of sense and antisense intronic miRNAs in *C. elegans *(31 versus 21, respectively). Later, the transcription of some sense-oriented intronic miRNAs is gradually switched from the intronic promoter to transcription from their host genes, the intronic promoter loses its function and the miRNAs becomes 'true' intronic miRNA. In the alternative scenario, some host gene-reliant miRNAs evolved in the intronic sequences *de novo *and were never transcribed from their own promoters; mirtrons are the ultimate example of such an evolutionary scenario [28]. Thus, two types of intronic miRNAs could be distinguished: true intronic miRNAs are processed as part of host gene transcripts and independent intronic miRNAs that reside in introns of genes in the sense orientation but can be transcribed from their own intronic promoters. These independent intronic miRNAs could also be processed from the host gene transcripts and, thus, the cumulative expression pattern of such miRNAs is probably composed of expression driven by the host gene promoter and the intronic miRNA promoter. Interestingly, processing of the mRNA-embedded hairpins by the Microprocessor complex has been recently recognized as an independent gene regulatory pathway [12], and investigation of the interplays between specific intronic miRNAs and their host genes would be a promising future direction in miRNA research.

## Conclusions

We have analysed the genomic environment of intronic miRNAs and found that almost half of the same-strand intronic miRNAs have long regions of extensive conservation immediately upstream of the pre-miRNAs. All of the seven tested conserved regions drive GFP expression in transgenic *C. elegans *and produce expression patterns that are different from the expression patterns of the host genes but are supported by the small RNA cloning data. Our results, combined with the previously published data for two additional intronic miRNAs, provide evidence for the presence of independent promoter regions for nine intronic miRNA genes and suggest that the fraction of intronic miRNAs that are transcribed independently from the host genes is higher than previously appreciated. The generated expression profiles of intronic miRNA promoters will be valuable for further studies of interactions between intronic miRNAs and their host genes.

## Materials and methods

### Construction of *Pmir*::*gfp *reporters

We used the UCSC genome browser [47] to determine the conserved regions upstream of the predicted stem-loop sequence of intronic miRNAs. These conserved regions were cloned as the promoter regions upstream of the *gfp *gene with let-858 3' untranslated region. The following *Pmir::gfp *constructs were generated by the restriction enzyme-based cloning (*Not*I and *Afl*II sites) into the pCFJ151-p5605 vector [48] that also contains *unc-119 *selection gene: *Pmir-58*, *Pmir-67*, *Pmir-71*, *Pmir-82*, *Pmir-86*, *Pmir-87 *and *Pmir-124*, *PY67D8A.1*, *Pppfr-1*, *PY56A3A.7 *and *Pkup-1*. The primers that were used for the amplification of the promoter sequences from the N2 genomic DNA are provided in Additional file 2.

### Generation and analysis of transgenic *C. elegans *lines

Transgenic *PmicroRNA*::*gfp *animals were generated by biolistic transformation of the DP38 (*unc-119(ed3)*) *C. elegans *strain as described previously [40], except *Ppprf-1*::*gfp *lines, which were generated by microinjection with *rol-*6 transformation marker [49]. One or several bombardments were performed for every construct until at least three independent transgenic lines were obtained. For every transgenic line, mixed populations of hermaphrodites were examined by fluorescence microscopy. We recorded the expression pattern conferred by each miRNA promoter that was consistent in each of the independently derived transgenic lines.

## Abbreviations

GFP: green fluorescent protein; mRNA: messenger RNA; miRNA: microRNA; nt: nucleotide; pre: precursor; priRNA: primary RNA.

## Competing interests

The authors declare that they have no competing interests.

## Authors' contributions

EB designed the experiment. MI generated constructs and transgenic lines. MI, HCK and EB analysed the data and wrote the manuscript.

## Supplementary Material

Additional file 1 Intronic miRNAs excluded from the promoter analysis. Click here for file

Additional file 2 Location and sequences of primers used to amplify promoter regions. Click here for file
