# Supplementary material for: Expression patterns of intronic microRNAs in Caenorhabditis elegans
Source: Silence. 2010 Feb 1;1:5. doi: 10.1186/1758-907X-1-5 (PMC2835999; doi:10.1186/1758-907X-1-5)
Supplement: Additional file 1 — Intronic miRNAs excluded from the promoter analysis. [file 1758-907X-1-5-S1.PDF]

Additional data file 1. Intronic miRNAs excluded from the promoter analysis

**miRNA**	**Host gene**	**Comment**
mir-50	Y71G12B.11	short distance from exon boundary: 120 nt
mir-62	T07C5.1	mirtrons
mir-70	T10H9.5	short distance from exon boundary: 88 nt
mir-233	*W03G11.4*	short distance from exon boundary: 185 nt
mir-243	R08C7.12	no conservation;<br>repeat sequence immediately upstream of pre-miRNA
mir-253	F44E7.5	short distance from exon boundary: 55 nt
mir-254	ZK455.2	short distance from exon boundary: 140 nt
mir-272	Y66A7A.8	no conservation, no cloning data;<br>overlaps with repeat annotation;<br>possibly not real miRNA
mir-273	E01F3.1	no conservation, no cloning data;<br>possibly not real miRNA
mir-353	D1007.12	short distance from exon boundary: 106 nt;<br>small RNA cloning pattern inconsistent with miRNA biogenesis;<br>possibly not real miRNA
mir-1018	Y59E1B.1	mirtrons
mir-1019	M04C9.5	mirtron
mir-1020	T16G12.1	mirtron
mir-1822	ZK84.2	short distance from exon boundary: 280 nt
mir-1828	T22A3.5	short distance from exon boundary: 180 nt
mir-1829a	K09A9.5	no conservation in upstream region
mir-1829b	F20D1.3	no conservation in upstream region
mir-1829c	F39B1.1	no conservation in upstream region
mir-1830	B0286.3	no conservation in upstream region
mir-1831	C15F1.5	small RNA cloning pattern inconsistent with miRNA biogenesis;<br>overlap with snRNA annotation;<br>possibly not real miRNA
mir-1832	C18D11.4	short distance from exon boundary: 201 nt
mir-1833	Y41E3.4	short distance from exon boundary: 150 nt
