# Supplementary material for: Expression patterns of intronic microRNAs in Caenorhabditis elegans
Source: Silence. 2010 Feb 1;1:5. doi: 10.1186/1758-907X-1-5 (PMC2835999; doi:10.1186/1758-907X-1-5)
Supplement: Additional file 2 — Location and sequences of primers used to amplify promoter regions. [file 1758-907X-1-5-S2.PDF]

Additional data file 2. Location and sequences of primers used to amplify promoter regions

Promoter	Chr	From	To	Size, nt	Strand	Primer sequences
mir-58	IV	3231600	3233251	1651	+	F: atacttaaggtaataccgaattttggctc<br>R: atagcggccgcatcgggggataggtcgagag
mir-67	III	5931400	5932207	807	-	F: atacttaaggtgagcttcgatactttttc<br>R: atagcggccgcagttggaataccagagaatt
mir-71	I	9380011	9382333	2322	-	F: atacttaagtgctcatcttgcacatctag<br>R: atagcggccgcaacctctgtgtgtgagctagtt
mir-82	X	2435336	2435919	583	-	F: atacttaagtttcgtcagctcctaatgg<br>R: atagcggccgcaaagaacctatttcatgagttcac
mir-86	III	11936617	11937723	1106	-	F: cgccttaaggtcaggcaggca<br>R: atagcggccgctgtctcttttaaagggacagg
mir-87	V	12038759	12040754	1995	-	F: atacttaaggtgagtagttttcaagataaatc<br>R: atagcggccgctgtgttgaggatgagaagtt
mir-124	IV	11870990	11871706	716	+	F: gaactgcagatctccccttccatgctcca<br>R: cgctctagaatggtgtatcaattgcgaagga
kup-1	V	12040851	12041566	715	-	F: gaccctnagcctgcagctgataaaactcagcattag<br>R: gacgcggccgcggatccgctgaaataaataaaatttag
Y56A3A.7	III	11946899	11947939	1040	-	F: gaccctnagcctgcagagcacataattttcagacac<br>R: gacgcggccgcggatcctctggaaaaattaaaagtta
Y67D8A.1	IV	3225309	3225606	297	+	F: gaccctnagcctgcagaaattctaaatttccaaaaaaaagcgg<br>R: gacgcggccgcggatccttcctggaaatttttagttttgg
ppfr-1	I	9389576	9390825	1250	-	F: ctgcttaagaacattgcaacagcaaaaag<br>R: gtgcgccggcgtccttcgccactatttgatc
